# The Vital Role of Public Health Nurses in Perinatal HIV Prevention and Elimination

**DOI:** 10.7759/cureus.38704

**Published:** 2023-05-08

**Authors:** Rajnandini Prasad, Arpita Jaiswal, Roshan Prasad, Mayur B Wanjari, Dr. Ranjana Sharma

**Affiliations:** 1 Department of Medicine, Jawaharlal Nehru Medical College, Datta Meghe Institute of Higher Education and Research, Wardha, IND; 2 Department of Obstetrics and Gynaecology, Jawaharlal Nehru Medical College, Datta Meghe Institute of Higher Education and Research, wardha, IND; 3 Department of Medicine and Surgery, Jawaharlal Nehru Medical College, Datta Meghe Institute of Higher Education and Research, Wardha, IND; 4 Department of Research and Development, Jawaharlal Nehru Medical College, Datta Meghe Institute of Higher Education and Research, Wardha, IND; 5 Department of Medical Surgical Nursing, Smt. Radhikabai Meghe Memorial College of Nursing, Datta Meghe Institute of Higher Education and Research, Wardha, IND

**Keywords:** barriers, interventions, public health nurses, elimination, prevention, perinatal hiv

## Abstract

Perinatal HIV transmission remains a significant public health challenge, with an estimated 160,000 children newly infected with HIV each year. Public health nurses play a critical role in the prevention and elimination of perinatal HIV transmission through targeted interventions such as identification of pregnant women with HIV, referral and linkage to care, provision of antiretroviral therapy, and follow-up and retention in care for both mothers and infants. However, significant barriers to successful implementation exist, including stigma and discrimination, limited access to healthcare services, socioeconomic factors, and limited resources. Addressing these barriers will require a multifaceted approach that includes policy changes, community engagement, and targeted support and resources for affected families. In this review article, we provide an overview of the epidemiology of perinatal HIV transmission, current strategies for prevention and elimination, and the vital role of public health nurses in these efforts. We will also discuss the barriers to the successful implementation of public health nurse interventions and the future directions for research and practice in this field. Ultimately, the goal of perinatal HIV prevention and elimination can only be achieved through a sustained and collaborative effort across multiple sectors and stakeholders, with public health nurses playing a crucial role in this effort.

## Introduction and background

Perinatal HIV transmission refers to transmitting the human immunodeficiency virus from a mother to her infant during pregnancy, labor, delivery, or breastfeeding. This transmission mode remains a significant public health concern worldwide, with devastating consequences for both the mother and the infant. In 2020, an estimated 150,000 children were newly infected with HIV globally, with sub-Saharan Africa accounting for most cases [1,2].

Perinatal HIV transmission can have serious health implications for both the mother and the infant, including increased morbidity and mortality, reduced quality of life, and increased healthcare costs. Infants who acquire HIV risk developing AIDS and other opportunistic infections, which can compromise their immune systems and lead to life-threatening illnesses. HIV-positive mothers may experience a range of health complications, including progressive immune system damage and an increased risk of developing AIDS-related illnesses [3,4].

Despite these challenges, there has been significant progress in perinatal HIV prevention and elimination efforts in recent years. The introduction of antiretroviral therapy (ART) has dramatically reduced the risk of mother-to-child transmission, with some studies reporting transmission rates as low as 0.5%. Additionally, public health interventions such as HIV testing, antenatal care, and prevention of mother-to-child transmission (PMTCT) programs have been successful in identifying and managing HIV-positive pregnant women and in providing appropriate care and treatment to ensure the health and safety of both the mother and the infant [5,6].

The vital role of public health nurses in perinatal HIV prevention and elimination cannot be overstated. Public health nurses play a critical role in identifying and managing HIV-positive pregnant women, providing appropriate care and treatment, and ensuring their infants are protected from HIV transmission. These nurses are often on the front lines of PMTCT programs, providing a range of services, including HIV testing, counseling, ART initiation and management, and follow-up and retention in care. Without the efforts of public health nurses, many women and infants would remain at risk of perinatal HIV transmission, with potentially devastating consequences for their health and well-being [7,8].

The objective of this review is to examine the importance of public health nurses in perinatal HIV prevention and elimination, as well as their key interventions and potential barriers to successful implementation. By highlighting the critical role of public health nurses in this area of healthcare, we hope to raise awareness of the ongoing challenges and opportunities in perinatal HIV prevention and elimination and inspire continued efforts to improve the health and well-being of affected families around the world.

## Review

Methodology

A comprehensive literature search was conducted using electronic databases such as PubMed, Medical Literature Analysis and Retrieval System Online (MEDLINE), PsycINFO, and the Cochrane Library. The search encompassed articles published from the year 2000 to the present. It utilized specific keywords such as "barriers", "interventions", "public health nurses", "elimination", "prevention," and "perinatal HIV." The articles were screened for relevance and eligibility based on inclusion and exclusion criteria. The inclusion criteria required that the articles be published in the English language, report on perinatal HIV transmission, associated factors, and potential health consequences, report on both observational and interventional studies published from the year 2000 to the present, and not be duplicates. The exclusion criteria required that the articles be published in non-peer-reviewed journals and published before 2000.

Perinatal HIV transmission

Perinatal HIV transmission, also known as mother-to-child transmission (MTCT) of HIV, is a major global public health issue that affects millions of people worldwide. According to the Joint United Nations Programme on HIV/AIDS (UNAIDS), an estimated 1.5 million pregnant women were living with HIV globally in 2020. Without intervention, up to 45% of their infants could become infected with HIV during pregnancy, delivery, or breastfeeding [9].

However, with the right interventions, the risk of perinatal HIV transmission can be reduced to less than 2% [3]. Current strategies for preventing and eliminating perinatal HIV transmission include a combination of biomedical, behavioral, and social interventions. These strategies include HIV testing and counseling for pregnant women and their partners, antiretroviral therapy (ART) for HIV-positive pregnant women, delivery by elective cesarean section (ECS) for women with high viral loads, and exclusive formula feeding for infants of HIV-positive mothers. These interventions are collectively known as the Prevention of Mother-to-Child Transmission (PMTCT) program [1,10].

Public health nurses play a critical role in the PMTCT program by identifying and managing HIV-positive pregnant women, ensuring their adherence to ART and other PMTCT interventions, and providing support and counseling throughout pregnancy and postpartum [11,12]. They also work with other healthcare providers and community partners to promote HIV testing, prevention, and treatment services and to address barriers to successful PMTCT implementation. By providing comprehensive and coordinated care to HIV-positive pregnant women and their infants, public health nurses can help reduce the risk of perinatal HIV transmission and improve health outcomes for affected families [13,14].

Public health nurse intervention in perinatal HIV prevention and elimination

Public health nurses play a vital role in preventing and eliminating perinatal HIV transmission through a range of interventions aimed at identifying and managing HIV-positive pregnant women, ensuring their adherence to antiretroviral therapy (ART) and other PMTCT interventions, and providing support and counseling throughout pregnancy and postpartum. Below are some of the key interventions carried out by public health nurses in this regard.

Identification of Pregnant Women With HIV

Identifying pregnant women with HIV is a critical first step in preventing perinatal HIV transmission. Public health nurses are often the first point of contact for pregnant women seeking healthcare services, whether through prenatal clinics, community outreach programs, or other healthcare settings. They play a critical role in identifying pregnant women living with HIV through routine HIV testing and counseling, partner notification and testing, and contact tracing [15,16].

Routine HIV testing during pregnancy is recommended by the World Health Organization (WHO) and the Centers for Disease Control and Prevention (CDC) to identify pregnant women who are living with HIV and who may not know their status. Public health nurses can offer HIV testing to pregnant women during their first prenatal visit and throughout their pregnancy, as national guidelines recommend. They can also provide pre-test and post-test counseling to help women understand the importance of testing and ensure that they receive appropriate care and support if diagnosed with HIV [4,14].

Partner notification and testing are important strategies for identifying pregnant women with HIV. Public health nurses can work with women to identify and notify their partners of their potential exposure to HIV and encourage them to seek testing and care. They can also offer testing and counseling to partners to help ensure that they receive appropriate care and support if they are diagnosed with HIV [1,2,17].

Finally, contact tracing is a strategy that can be used to identify pregnant women who may have been exposed to HIV through other means, such as needle-sharing or unprotected sex. Public health nurses can work with women to identify potential sources of exposure and offer testing and counseling to ensure that they receive appropriate care and support if they are diagnosed with HIV [8].

Referral and Linkage to Care for HIV-Positive Pregnant Women

Referral and linkage to care for HIV-positive pregnant women are critical in preventing perinatal HIV transmission. Public health nurses are crucial in facilitating this process by ensuring that HIV-positive pregnant women are promptly referred to appropriate HIV care and treatment services. This includes providing education and support to help women understand the importance of ART and PMTCT interventions and addressing any concerns or barriers to care that may exist [18,19].

Public health nurses work closely with HIV care and treatment providers to ensure pregnant women receive timely and appropriate care. They may also provide additional support and counseling to help women navigate the healthcare system and understand the importance of adhering to their treatment regimen. By facilitating referral and linkage to care, public health nurses can help ensure that HIV-positive pregnant women receive the care they need to protect their health and the health of their infants [3,15,18].

In addition, public health nurses may also work to address social and economic barriers to care that may prevent HIV-positive pregnant women from accessing care. For example, they may provide transportation assistance, assist with navigating insurance and financial assistance programs, or connect women to community resources that can help address their basic needs. By addressing these barriers, public health nurses can help ensure that HIV-positive pregnant women receive the care and support they need to prevent perinatal HIV transmission and promote optimal health outcomes for themselves and their infants [7,13,16].

Antiretroviral Therapy for HIV-Positive Pregnant Women

Antiretroviral therapy (ART) is critical to perinatal HIV prevention and elimination. Public health nurses are important in ensuring that HIV-positive pregnant women receive ART according to national guidelines. This typically involves working with healthcare providers to initiate ART as soon as possible after diagnosis and continuing treatment throughout pregnancy and postpartum [20]. ART helps suppress the viral load and reduce the risk of mother-to-child transmission of HIV. Public health nurses may also provide education and counseling to HIV-positive pregnant women about the importance of ART adherence and the potential benefits and risks associated with ART use during pregnancy and postpartum. In addition, public health nurses may work with healthcare providers to monitor the effectiveness of ART and adjust treatment as necessary to ensure optimal outcomes for both mother and child. By working collaboratively with healthcare providers, public health nurses can help ensure that HIV-positive pregnant women receive timely and effective ART, which is critical for preventing and eliminating perinatal HIV transmission [21].

Prevention of Mother-to-Child Transmission (PMTCT) Interventions

Public health nurses are critical to preventing mother-to-child transmission (PMTCT) of HIV. PMTCT interventions involve a comprehensive approach that includes prophylaxis with antiretroviral drugs, delivery by elective cesarean section (ECS) for women with high viral loads, and exclusive formula feeding for infants of HIV-positive mothers. Public health nurses work closely with HIV-positive pregnant women to ensure they receive appropriate PMTCT interventions and understand the importance of adherence to these interventions to reduce the risk of transmission to their infants [22].

In addition to providing prophylaxis and other medical interventions, public health nurses also provide counseling and support to promote adherence to these interventions and address any concerns or barriers that may arise. This may include counseling around the importance of adhering to antiretroviral therapy (ART), ensuring that infants receive formula exclusively, and encouraging women to have an elective cesarean section if indicated. Public health nurses may also provide emotional support and referrals to additional services as needed, such as mental health counseling or social services [23,24].

Follow-Up and Retention in Care for HIV-Positive Pregnant Women and Their Infants

Follow-up and retention in care for HIV-positive pregnant women and their infants are critical components of perinatal HIV prevention and elimination. Public health nurses are critical in ensuring HIV-positive pregnant women and their infants remain engaged in care and receive appropriate follow-up services after delivery [1,9,20]. To achieve this, public health nurses must monitor the adherence of HIV-positive pregnant women to ART and PMTCT interventions, provide counseling and support, and refer patients to additional services as needed. In addition, public health nurses must work with healthcare providers and community organizations to ensure that HIV-positive pregnant women and their infants receive appropriate care and support throughout the perinatal period and beyond [18,21].

Effective follow-up and retention in care require a comprehensive approach that includes ongoing monitoring and support, education and counseling, and access to appropriate healthcare services. Public health nurses can play a critical role in facilitating this approach by working with healthcare providers, community organizations, and families to develop personalized care plans that address HIV-positive pregnant women's and their infants' unique needs. This may include providing ongoing support and counseling, monitoring adherence to medication and follow-up appointments, and connecting families to additional resources and services as needed [10,12].

Ultimately, the success of follow-up and retention in care efforts will depend on the ability of public health nurses to build strong relationships with patients and families, develop a deep understanding of the unique challenges each patient faces, and provide tailored support and resources to meet their individual needs. By doing so, public health nurses can help ensure that HIV-positive pregnant women and their infants receive the care and support they need to lead healthy, productive lives [20,23,25].

Partner Testing and Linkage to Care

Partner testing and linkage to care are critical components of perinatal HIV prevention and elimination. Public health nurses recognize the important role that partners play in the lives of pregnant women and their families, and they understand that partner testing and treatment are essential for reducing the risk of HIV transmission within the household and community [1,17]. Public health nurses work closely with HIV-positive pregnant women to encourage their partners to get tested for HIV and to support them in accessing appropriate care and treatment services if needed. They may provide education and counseling to both the pregnant woman and her partner on the importance of HIV testing and treatment and strategies for reducing the risk of HIV transmission. This may include promoting condoms, providing information on PrEP, and encouraging regular HIV testing and check-ups. By engaging partners in the PMTCT process, public health nurses can help create a supportive and empowering environment that fosters optimal health outcomes for all household members. Ultimately, partner testing and linkage to care aim to help prevent new HIV infections and ensure HIV-positive individuals have access to the care and treatment they need to live healthy and fulfilling lives [12,21,23].

Barriers to the successful implementation of public health nurse intervention

While public health nurses play a critical role in the prevention and elimination of perinatal HIV transmission, several barriers can limit their ability to implement interventions successfully. These barriers can be categorized into several broad categories, including stigma and discrimination, limited access to healthcare services, socioeconomic factors, and limited resources. Below are some examples of each.

Stigma and Discrimination

Stigma and discrimination related to HIV can significantly negatively impact HIV testing, treatment, and care, particularly among pregnant women and their partners. For example, HIV-related stigma and discrimination may discourage people from getting tested for HIV, seeking treatment, or adhering to medication regimens. This can lead to delayed diagnoses, decreased treatment adherence, and increased rates of perinatal HIV transmission.

Public health nurses who work with pregnant women with HIV may also encounter stigma and discrimination in the course of their work. This can come from various sources, including healthcare providers who may hold biased beliefs about people living with HIV, community members who may stigmatize pregnant women with HIV, and even the women they serve themselves, who may internalize societal stigma around HIV. When public health nurses encounter stigma and discrimination in their work, engaging patients in care and providing effective support and counseling can be difficult.

To address these challenges, public health nurses can take several steps to reduce stigma and discrimination in healthcare settings. This may include providing education and training to healthcare providers, advocating for policy changes that protect the rights of people living with HIV, and working with community organizations to raise awareness about HIV and reduce stigma. In addition, public health nurses can provide emotional and social support to their patients to help them cope with the challenges of living with HIV and build social networks that can help reduce isolation and stigma. Overall, addressing stigma and discrimination is critical to the success of perinatal HIV prevention and elimination efforts and must be a key focus of any comprehensive public health strategy aimed at addressing the HIV epidemic [26-28].

Limited Access to Healthcare Services

Limited access to healthcare services can be a significant barrier to successful PMTCT implementation. HIV-positive pregnant women require timely and high-quality healthcare services to prevent mother-to-child transmission of the virus. However, many pregnant women and their families may face significant challenges in accessing these services, which can impact the effectiveness of PMTCT interventions [29].

Geographic and transportation barriers can make it difficult for pregnant women to travel to healthcare facilities, particularly in rural or remote areas where services may be limited. Financial constraints can also pose significant challenges, particularly for low-income women who may struggle to afford transportation, medications, or other healthcare-related expenses [25,30].

Language and cultural barriers can also impact access to care, particularly for immigrant or refugee populations who may have limited proficiency in the local language or cultural norms that differ from those in their home countries. These barriers can make it difficult for public health nurses to identify and manage HIV-positive pregnant women, provide appropriate counseling and support, and ensure their adherence to ART and PMTCT interventions [30].

Addressing these barriers will require a multifaceted approach that includes improving access to healthcare services through expanded service delivery, transportation subsidies, and other support programs. This may also require targeted interventions that address the specific needs of different populations, such as language and cultural support for immigrant and refugee populations. Public health nurses can play a key role in identifying and addressing these barriers through community engagement and outreach, advocacy efforts, and targeted support and resources for affected families [19,25,30].

Socioeconomic Factors

Socioeconomic factors can have a profound impact on the successful implementation of PMTCT interventions. For instance, pregnant women living in poverty, experiencing unemployment, or struggling with housing instability may find it challenging to prioritize their healthcare needs, including accessing PMTCT services. These factors can create additional stressors and challenges for pregnant women, which may lead to delays in accessing care, missed appointments, and poor adherence to treatment regimens [31,32].

Public health nurses may need to provide additional support and resources to help address these socioeconomic barriers and ensure that pregnant women have the necessary resources to access care. This may include helping women connect with social service agencies and providing transportation [33].

Limited Resources

Limited resources can be a major impediment to the successful implementation of perinatal HIV prevention and elimination programs. These resources may include human and financial resources, such as staffing and budget constraints, as well as inadequate infrastructure and equipment. When public health nurses are working with limited resources, it can have a negative impact on their ability to effectively identify and manage HIV-positive pregnant women and provide appropriate support and counseling [12,23]. For example, public health nurses may have limited time to dedicate to each patient, which can make it difficult to provide comprehensive counseling and support. Additionally, limited resources may mean that public health nurses are unable to provide regular follow-up care and support to patients, which can lead to poor health outcomes and an increased risk of perinatal HIV transmission. Addressing these resource constraints will require a concerted effort from policymakers, healthcare organizations, and other stakeholders to ensure adequate funding and resources are allocated to support effective PMTCT programs. This may include investing in staff training and development, improving healthcare infrastructure, and increasing funding for PMTCT interventions. By addressing these resource constraints, public health nurses can improve their ability to provide effective care and support to HIV-positive pregnant women and their families and ultimately help prevent perinatal HIV transmission [7,11,16,19,30].

## Conclusions

Public health nurses play a vital role in the prevention and elimination of perinatal HIV transmission. Through targeted interventions such as identification of pregnant women with HIV, referral and linkage to care, provision of ART, and follow-up and retention in care for both mothers and infants, public health nurses can help reduce the risk of transmission and improve health outcomes for affected families. However, significant barriers to successful implementation exist, including stigma and discrimination, limited access to healthcare services, socioeconomic factors, and limited resources. Addressing these barriers will require a multifaceted approach that includes policy changes, community engagement, and targeted support and resources for affected families. Looking forward, future research should focus on identifying effective strategies for addressing these barriers and improving the effectiveness of PMTCT interventions. This may include exploring new models of care delivery, leveraging technology to improve access to care, and developing interventions that address the complex socioeconomic factors that contribute to HIV transmission. In addition, ongoing advocacy and policy efforts will be critical to ensuring that PMTCT programs are adequately resourced and supported. Ultimately, the goal of perinatal HIV prevention and elimination can only be achieved through a sustained and collaborative effort across multiple sectors and stakeholders.
